# Assessment of a Semi-solid Extrusion Based Compounding System Solution for Personalized Ondansetron Dosage Forms Combined with Raman Spectroscopy Analysis

**DOI:** 10.1007/s11095-025-03911-6

**Published:** 2025-08-19

**Authors:** Mahsa Bahman, Jacopo Zini, Julius Lahtinen, Niko Hassinen, Soumya Verma, Timo Laaksonen, Sari Airaksinen, Niklas Sandler Topelius, Tapani Viitala

**Affiliations:** 1 Pharmaceutical Sciences Laboratory, Åbo Akademi University, Artillerigatan 6A, 02520 Turku, Finland; 2 CurifyLabs Oy, Salmisaarenaukio 1, 00180 Helsinki, Finland; 3 Drug Research Program, Division of Pharmaceutical Biosciences, Faculty of Pharmacy, University of Helsinki, Viikinkaar, 5E 00790 Helsinki, Finland; 4 Drug Research Program, Division of Pharmaceutical Chemistry and Technology, Faculty of Pharmacy, University of Helsinki, Viikinkaari, 5E 00790 Helsinki, Finland

**Keywords:** ondansetron, personalized medicine, PLS model, quality control, raman spectroscopy

## Abstract

**Objective:**

3D printing and extrusion-based technologies, especially semi-solid extrusion (SSE), are promising solutions to fulfil the need to personalize pediatric medicines. In this study an automated SSE based Compounding System Solution (CSS) technology was assessed for creating customized Ondansetron tablets. Additionally, a non-destructive quality control method for the customized Ondansetron tablets was developed by utilizing Raman Spectroscopy (RS) measurements and partial least square (PLS) analysis.

**Methods:**

Tablets of 400 mg with varying Ondansetron content (2–10 mg) and different sizes (200–500 mg) with 0.5% Ondansetron were manufactured and tested according to European and US Pharmacopoeia standards, HPLC, and the RS-based PLS model.

**Results:**

The mass uniformity tests showed high accuracy: 99.2% for varying drug content and 98.8% for different tablet sizes. All tablets met the acceptance criteria (AV < 15) and remained stable for six months at 25 ± 2 °C and ambient humidity. *In-vitro* dissolution tests confirmed over 85% drug release within 30 min, complying with USP standards. The RS-based PLS model predicted active pharmaceutical ingredient (API) content with a slope of 0.944 and an error of ~ 8%, which improved to 2–3% when excluding highly variable 10 mg samples. The model showed strong correlation with HPLC results and prediction (R^2^CV = 0.95, RMSECV = 0.68; R^2^Pred = 0.96, RMSEP = 0.57), using three latent variables.

**Conclusion:**

In conclusion, the CSS technology, validated through pharmacopoeia tests, HPLC, and RS, effectively produces high-quality, personalized Ondansetron tablets. The study demonstrates the feasibility of using SSE and RS-based quality control for individualized pediatric drug formulations.

**Graphical Abstract:**

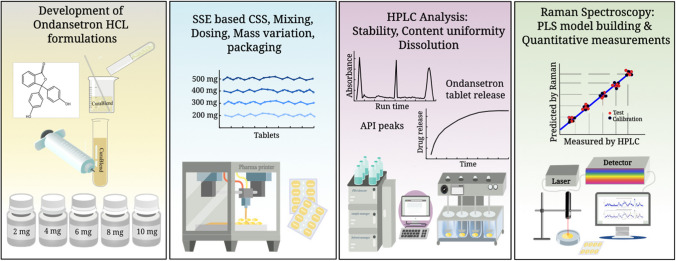

**Supplementary Information:**

The online version contains supplementary material available at 10.1007/s11095-025-03911-6.

## Introduction

Premature infants and pediatric patients have limited pharmaceutical treatment options. Despite numerous studies and efforts by health authorities, many medications are not available for pediatric patients [1]. Moreover, because of lack of appropriate dosage forms for pediatric patients, most oral drugs are developed in liquid form or suspensions, which often suffer from poor stability, unpleasant taste, and high production costs [2]. Most pharmaceutical companies seem to consider the pediatric and neonatal drug market as a non-lucrative segment, thus their target populations focus on adults.

In 2015, the first 3D printed tablet, Spritam® was approved by US Food and Drug Administration (FDA) for epilepsy treatment which opened new opportunities for personalized pharmaceutical manufacturing [3]. In recent decades, 3D printing, and related technologies have shown promise in addressing the needs of pediatric patients. However, using a layer-by-layer approach is time-consuming and cost-ineffective [4, 5]. Semi-solid extrusion (SSE) utilizes semi-solid or semi-molten materials. Whereas other 3D printing methods such as Fused Deposition Modeling (FDM) [6, 7] and Direct Powder Extrusion (DPE) [8, 9] require solid polymer filaments or powders as starting materials, respectively. FDM and DPE face challenges in pharmaceutical 3D printing due to their high-temperature processes (i.e., temperature typically higher than 120 °C), which can degrade heat-sensitive drugs and limit material compatibility, decrease drug stability, and cause filament aging. Selective Laser Sintering (SLS) is an advanced 3D printing technique that offers several advantages, including high resolution, solvent-free processing, and process repeatability. However, a significant concern, together with lack of practicality, in pharmaceutical applications is the potential degradation of APIs due to exposure to the high-energy laser beam [10].

In contrast, the SSE technique uses mostly semi-solid pastes, i.e., gelatin, agar, hydrogel etc., allowing the drug to remain chemically stable during both the printing process and long-term storage. Among all 3D printing techniques, SSE was reported as the most used approach, accounting for 23% of the studies published in the past decade [10]. SSE operates at low temperatures, i.e., around room temperature to slightly elevated levels (up to ~ 75 °C) [11], making it more suitable for personalized medicines that are sensitive to high printing temperature or UV light/beams [5]. SSE also offers formulation flexibility, enabling the incorporation of hydrophilic polymers and bio adhesive excipients ideal for orodispersible dosage forms, which are preferred for patients with nausea. Moreover, SSE ensures precise dosing, making it highly suitable for personalized medicine applications in sensitive populations within the areas of pediatrics and geriatrics [12].

SSE-based technologies offer the choice of manufacturing of a wide range of dosage forms, shapes, colors, flavors and different base formulations [13–16]. In general, the printability in semisolid extrusion 3D printing is influenced by several factors, including the ink formulation, the heating process, the material and type of the printhead or syringe, as well as the physical and chemical properties of the formulation and the API content. Recent research has provided valuable insights into how ink formulation components, specifically HPMC (Hydroxypropyl Methylcellulose), PVP (Polyvinylpyrrolidone), and SiO_2_ (Silicon Dioxide) affect the printability, structural fidelity, and drug release performance of tablets produced via SSE 3D printing [17]. Additionally, recent research demonstrated that using reusable stainless-steel cartridges in SSE improves temperature control, reduces waste, and enhances formulation stability under thermal stress [18]. In a recent study, the application of SSE tablets in neonates has been emphasized [19]. This study focuses on semi-solid extruded tablets for personalized pediatric use and highlights their suitability for nasogastric tube (NGT) administration, a critical route for children who cannot swallow solid dosage forms. The tablets, designed using SSE 3D printing, demonstrated excellent disintegration and dispersion properties, ensuring they could be smoothly and safely delivered through NGTs without clogging or compromising drug content uniformity.

Prior to the introduction of the European Pediatric Regulation (EC) No 1901/2006, there was limited emphasis on developing age-appropriate medicines for pediatric patients [20]. Among all medicines for pediatric patients, Ondansetron is one of the important API that requires personalization because of its unpleasant taste and serious side effects if dosage does not meet the need of the patients. Moreover, due to the wide variability in the age of children, such as in body size and metabolic rates, adjusting the Ondansetron content alongside co-administered therapies (such as cytotoxic drugs) is complex, which highlights the necessity for flexible and precise dosage forms [21]. Ondansetron is used alone or with other medications to prevent nausea and vomiting caused by cancer drug treatment (chemotherapy), radiation therapy or after surgery [22]. It works by blocking the 5-HT3 receptor (serotonin) that causes vomiting and nausea. However, this drug requires high quality control monitoring to avoid arrhythmia and QT-interval elongation in patients [23]. Ondansetron has previously been compounded as orally disintegrating tablets by using selective laser sintering (SLS) 3D printing to produce high quality dose-specific dosage forms [12].

Raman Spectroscopy (RS) has been used as a quality control tool in the pharmaceutical field to demonstrate drug quantification by using multivariate regression models, such as PLS (partial least squares) or PCA (Principal Component Analysis). In a previous study, RS data obtained from atorvastatin and lovastatin were used to build a PLS model aimed at drug quantification [24]. The RS technique has also been applied to 3D-printed medicines to predict amorphous content, both qualitatively and quantitatively, by developing calibration models via PLS regression [25, 26]. In a recent study, RS was applied as a mapping technique to demonstrate drug distribution and quantification in expired and non-expired drug formulations [27].

**Fig. 1:**
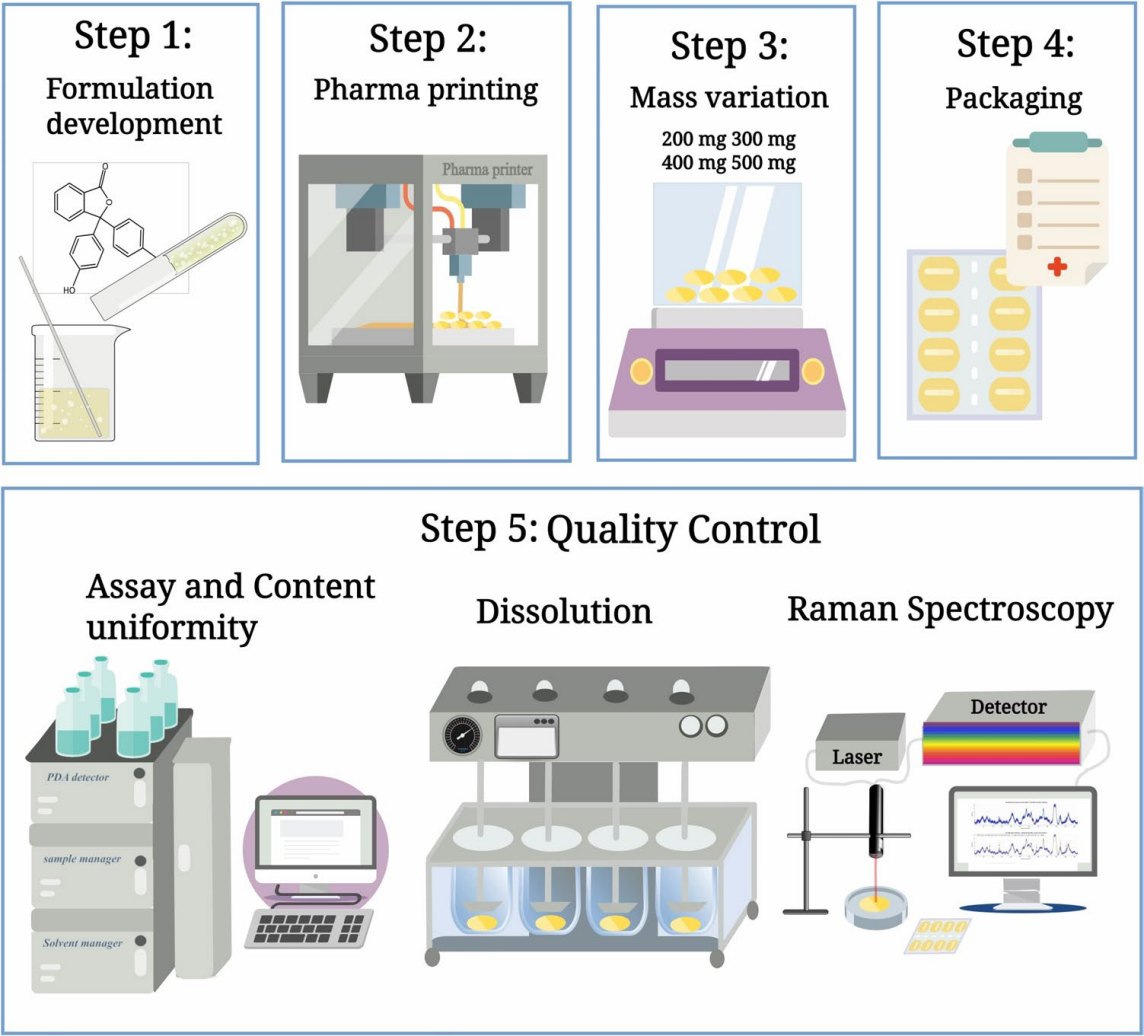
Step 1: Different contents of Ondansetron were prepared in a gel based (CuraBlend® gel tablet base) formulation. Step 2: The prepared formulations were mixed and dosed with the CSS technology. Step 3: Mass variation tests were performed to study the accuracy of the CSS technology. Step 4: The tablets were packed in blisters and stored for quality control measurements. Step 5: Quality control analyzes were done by HPLC (drug stability, content uniformity, *in-vitro* dissolution) and RS (quantitative analysis of Ondansetron formulations).

The aim of this study is to investigate and assess a SSE-based compounding technology for producing personalized drug dosage forms tailored for pediatric patients. The research focused on addressing the challenges associated with Ondansetron tablets, particularly the need for low-dose formulations. Comprehensive quality control evaluation, including assessments of drug uniformity, long-term stability, and *in-vitro* drug release using HPLC, were conducted. More importantly, the study investigated the application of RS as a non-destructive and rapid analytical method for ensuring the quality and safety of medicines, thereby proposing it as a viable alternative to traditional chromatographic techniques as a QC tool for drug quantification. Figure 1 summarizes the workflow for this study.

## Materials & Methods

### Pharma Ink Preparation

Ondansetron hydrochloride dihydrate (hereafter referred to as Ondansetron) from Dr. Reddy's Laboratories (Hyderabad, India) was formulated at different strengths of 0.5, 1, 1.5, 2, and 2.5% in a gelatin-based formulation (CuraBlend® gel tablet base, CurifyLabs Oy, Helsinki, Finland), with the addition of polysorbate 80 as a surfactant (Caesar & Loretz GmbH, Hilden, Germany), as presented in Table I.

**Table I:** Ondansetron Formulations

Formulations	Ondansetron (%w/w)	Polysorbate 80 (%w/w)	CuraBlend® gel tablet base (%w/w)
0.5% Ondansetron	0.5	1	98.5
1% Ondansetron	1	1	98
1.5% Ondansetron	1.5	1	97.5
2% Ondansetron	2	1	97
2.5% Ondansetron	2.5	1	96.5

To prepare the formulations, the required amount of Ondansetron and 1%w/w polysorbate 80 were first weighed. The appropriate quantity of CuraBlend® gel tablet base was then added as the excipient base. Subsequently, all ingredients were mixed using a PM 140 planetary mixer (Gako Deutschland GmbH, Scheblitz, Germany) to ensure formulation homogeneity.

### Automated CSS Dosing Technology

The automated Compounding System Solution (CSS) is a pharmaceutical compounding technology that includes excipient bases (pharma inks), a mixer for incorporating the API and additional excipients into the base, semi-solid extrusion of pre-heated formulations, packaging, control software, and quality control analysis. Mixing of the excipients and the API was performed by using a PM 140 planetary mixer (Gako Deutschland GmbH, Scheblitz, Germany) at 2800 rpm for 2 min. The SSE-based Pharma Printer (CurifyLabs Oy, Helsinki, Finland) was used to dose the formulations with a printing temperature set to 41 °C for the CuraBlend® gel tablet base. Sterile, single-use 100 mL PVC syringes with a Luer-Lock mechanism (CurifyLabs Oy, Helsinki, Finland) were used as formulation cartridges. An analytical balance (Kern PEJ-620-3 M, Bellingen, Germany) was integrated into the Pharma Printer to measure and record the weight of the extruded formulations. The API content, formulation type, tablet size, and number of tablets were selected via custom software (V2.0, CurifyLabs Oy, Helsinki, Finland), which also assigned a specific order ID. Based on the input order, the printer initiated semi-solid extrusion of the formulations into blisters (3/16-inch Mini Medi-Cap® Plus™ Blisters, MD425, MediDose Inc., Ivyland, PA, USA), where the tablets formed upon cooling. The blisters were then manually sealed using the Fil-Form Template and Roll-E-ZY Press-Piece “25” (MediDose Inc., Ivyland, PA, USA).

### Mass Uniformity Test and Dosing Accuracy

Dosing accuracy was defined as the percentage of dosage units within a batch that met specified mass variation criteria. Mass uniformity tests were conducted by printing 200, 300, 400, and 500 mg tablets containing 0.5% Ondansetron. A total of 48 tablets were printed for each tablet size to evaluate the effect of tablet size on printing accuracy using a constant API concentration. Additionally, to assess the impact of increasing Ondansetron content on mass uniformity, 400 mg tablets containing 2, 4, 6, 8, and 10 mg of Ondansetron were printed. The integrated balance into Pharma Printer was used to record the weight of each tablet during printing. The custom-made software includes a built-in quality control feature that flags any tablet whose weight deviates by more than ± 5% for tablets over 250 mg, and ± 7.5% for tablets under 250 mg, in accordance with Ph. Eur. Chapter 2.9.5 Uniformity of Mass of Single-Dose Preparations [28]. The same series of 400 mg tablets containing 2, 4, 6, 8, and 10 mg of Ondansetron were also prepared for RS analysis to represent the same range of drug content.

### Calculation of Weight and Size Variations

Ten tablets were randomly selected to assess size and weight variation. The tablets were weighed using the integrated balance of the Pharma Printer. The diameter and thickness of each tablet were measured manually using a Vernier caliper (Biltema, Linköping, Sweden).

### Determination of Content Uniformity

Content uniformity testing was performed using a validated HPLC method (see Supplementary Materials for detailed HPLC condition) by preparing 10 tablets of each size (200, 300, 400, and 500 mg) containing 0.5% Ondansetron. Additionally, API uniformity tests were conducted on 400 mg tablets containing 2, 4, 6, 8, and 10 mg of Ondansetron to assess content uniformity at higher drug concentrations. Each tablet was dissolved in a appropriate volumetric flask based on drug strength (final sample concentration was 50 µg/ml) and water was added to the mark. The samples were placed in a water bath at 50 °C for 10–15 min and mixed thoroughly to ensure homogeneity. After cooling to room temperature, the solutions were filtered through 0.22 µm membrane filters (MontaMil®, Frisenette ApS, Knebel, Denmark) to obtain clear samples. The API content of the samples was analyzed by HPLC. The Acceptance Value (AV) for content uniformity of 10 tablets was calculated according to Ph. Eur. guidelines [29]. An HPLC (Thermo Scientific™ Vanquish system, Germering, Germany) with the Chromeleon™ Chromatography Data System software (Dionex Softron GmbH, Germering, Germany) was utilized for sample analysis. The HPLC system was equipped with a C18 column (4.6 × 100 mm i.d., 2.5 μm particle size, VanGuard FIT, Wilmslow, UK) and Diode Array Detectors (Thermo Scientific Vanquish detector, Dionex Softron GmbH, Germering, Germany).

### Determination of Stability at Ambient Conditions

Stability tests were conducted on 400 mg tablets containing 0.5% Ondansetron in CuraBlend® gel tablet base. Six individual samples were prepared in 100 mL flasks for testing. Long-term stability studies were carried out under ambient conditions (20–25 °C, ambient humidity). Chemical stability (assay), visual evaluation of the tablets, and pH measurements were performed after 0, 1, 3, and 6 months.

### pH Determination

The pH of the formulation containing 0.5% Ondansetron CuraBlend® gel tablet base was measured at room temperature using a calibrated pH meter (HANNA Instruments, Inc., Woonsocket, USA). For each time point, a sample of the semi-finished product was melted at 50 °C to ensure consistency. Once liquefied, the pH electrode was immersed into the sample, and the pH value was recorded after stabilization of the reading. Measurements were conducted at 0, 1, 3, and 6 months of storage to evaluate the pH stability of Ondansetron within the formulation over time.

### *In Vitro *Dissolution

Dissolution studies were carried out using a Type II dissolution apparatus (DT 128 light; ERWEKA, Heidenstam, Germany) operated at 50 rpm and maintained at 37 ± 0.5 °C. Six tablets were placed in separate vessels containing 500 mL of deionized water as the dissolution medium. Samples of 1 mL were withdrawn at 5, 10, 15, 20, 30, 45, and 60 min from each vessel, with an equal volume of fresh deionized water added to compensate for sampling loss. The collected samples were then filtered through 0.22 μm PTFE membrane syringe filters and analyzed using HPLC.

### Quantitative Evaluation of Ondansetron Tablets Via RS

A Timegate PicoRaman M2 (Timegate Instruments, Oulu, Finland) equipped with a 532 nm pulse laser (150 ps pulse width) and a complementary metal–oxide–semiconductor single-photon avalanche diode (CMOS-SPAD) was used for the RS measurements. The spectrometer has a spatial resolution of 6 cm^−1^ and a temporal resolution of 150 ps. The tablets were measured using a non-contact BwTec probe (Delaware, USA). Each sample was measured five times with a laser power of ~ 60mW and a three-minute exposure time for each measurement. During the measurements the tablets were rotated under the laser to expose a wider area.

### Raman Data Analysis

A total of 65 semi-solid extruded tablets with an Ondansetron target of 2, 4, 6,8 and 10 mg of Ondansetron, i.e., 13 tablets for each strength were measured. Each tablet was measured five times and the average of these 5 measurements was used as the Raman spectrum. The pre-processing of the data was done by using custom-made MATLAB scripts (MathWorks, USA), while PLS analysis was performed using PLS_Toolbox (Eigenvector Research, Inc. WA, USA). The analysis of the Raman data proceeded as following. First, the averaged spectra were smoothed by Savitzky-Golay filter with 30 Raman shifts as the window, then the baseline was estimated by Asymmetric least-squares smoothing and mean centered before PLS analysis. The preprocessing steps of Raman spectra, variable selection and outplayed handling are shown in the supplementary material Figures S1-S3. Two outlier samples were removed from the test set due to high Hotelling's T^2^ and residual (Supplementary material, Figure S3). A continuous block with a block size of 10 measurements was used as Cross-validation (CV) for the PLS regression analysis. To validate the model, the dataset of 65 samples was divided in two sets: a training set, with 50 samples and a test set with 15 samples. PLS was run on 3 components based on the coefficient of determination (R^2^, Eq. 1) and Root Mean Square Error (RMSE, Eq. 2) as reported in the supplementary material (Figures S4-S5). Bias (Eq. 3) reflects the systematic error, while RMSE include both the systematic and random error.

1 $${R}^{2}=\frac{SSR}{SST}$$

2 $$RMSE=\sqrt{\frac{\sum_{i=1}^{n}{\left({\widehat{y}}_{i}-{y}_{i}\right)}^{2}}{n}}$$

3 $$Bias=\frac{\sum_{i=1}^{n}{\left({\widehat{y}}_{i}-{y}_{i}\right)}}{n}$$

SSR = is the sum of squared regression,

SST = the sum of squared total.

ŷ = predicted variable.

y = observed variable.

n = number of observations.

### Statistical Analysis

All statistical analyses were conducted using IBM SPSS Statistics software, version 27 (IBM Corp., Armonk, NY, USA). To evaluate differences among experimental groups, a one-way analysis of variance (ANOVA) was performed. Tukey`s Post Hoc test was conducted to compare mean differences between groups. Statistical significance was determined at a threshold of p < 0.05.

## Results

### Mass Uniformity and Weight Accuracy Tests

**Fig. 2:**
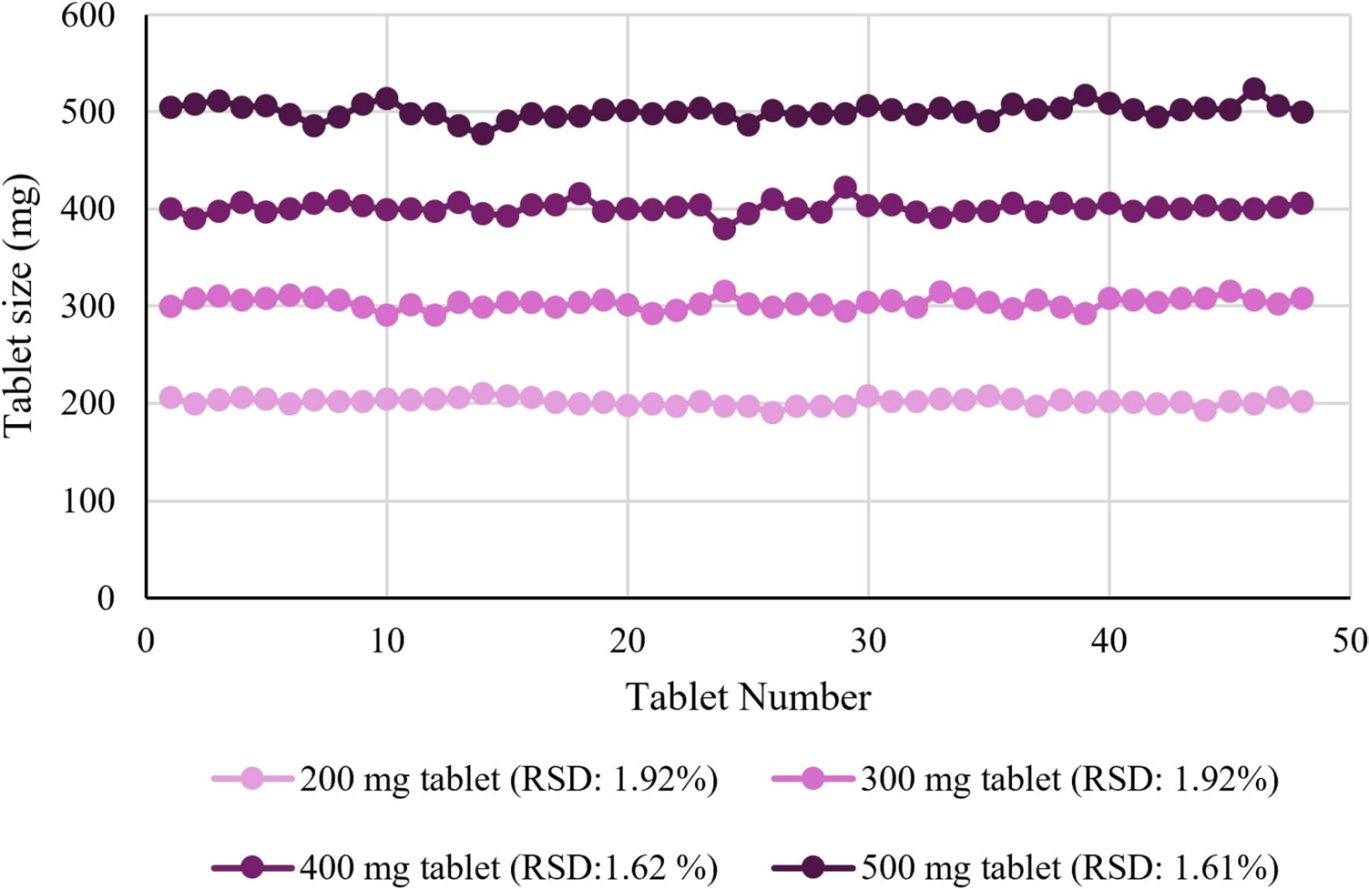
Mass uniformity test for 0.5% Ondansetron tablets

The mass uniformity results for 200, 300, 400, and 500 mg tablets indicated that the dosing accuracy averaged over 96% (Table II and Fig. 2). Moreover, Table II shows the minimum and maximum weights, as well as the diameter, and the thickness of semi-solid extruded 0.5% Ondansetron tablets. The Tukey`s Post Hoc test demonstrated that there were no statistically significant differences in mass uniformity or printing accuracy among the different tablet sizes of 200, 300, 400, and 500 mg (P > 0.05 with the smallest p-value = 0.10 observed between 500 and 300 mg).

**Table II:** Target API Content, Mass, Diameter, and Thickness Variation Data for 0.5% Ondansetron Tablets

weight (mg)	No. tablets	Target API content (mg)	Minimum (mg)	Maximum (mg)	Mean (mg)	Weight RSD (%)	Diameter (mm)	Diameter RSD (%)	Thickness (mm)	Thickness RSD (%)	Printing accuracy
200	48	1	190	207	201	1.92	12.71	3.14	1.73	2.34	100%
300	48	1.5	290	315	303	1.92	15.62	1.61	1.64	1.87	100%
400	48	2	379	422	401	1.62	17.78	1.19	1.72	1.56	96%
500	48	2.5	477	523	501	1.61	20.32	0.88	2.03	1.16	99%

**Fig. 3:**
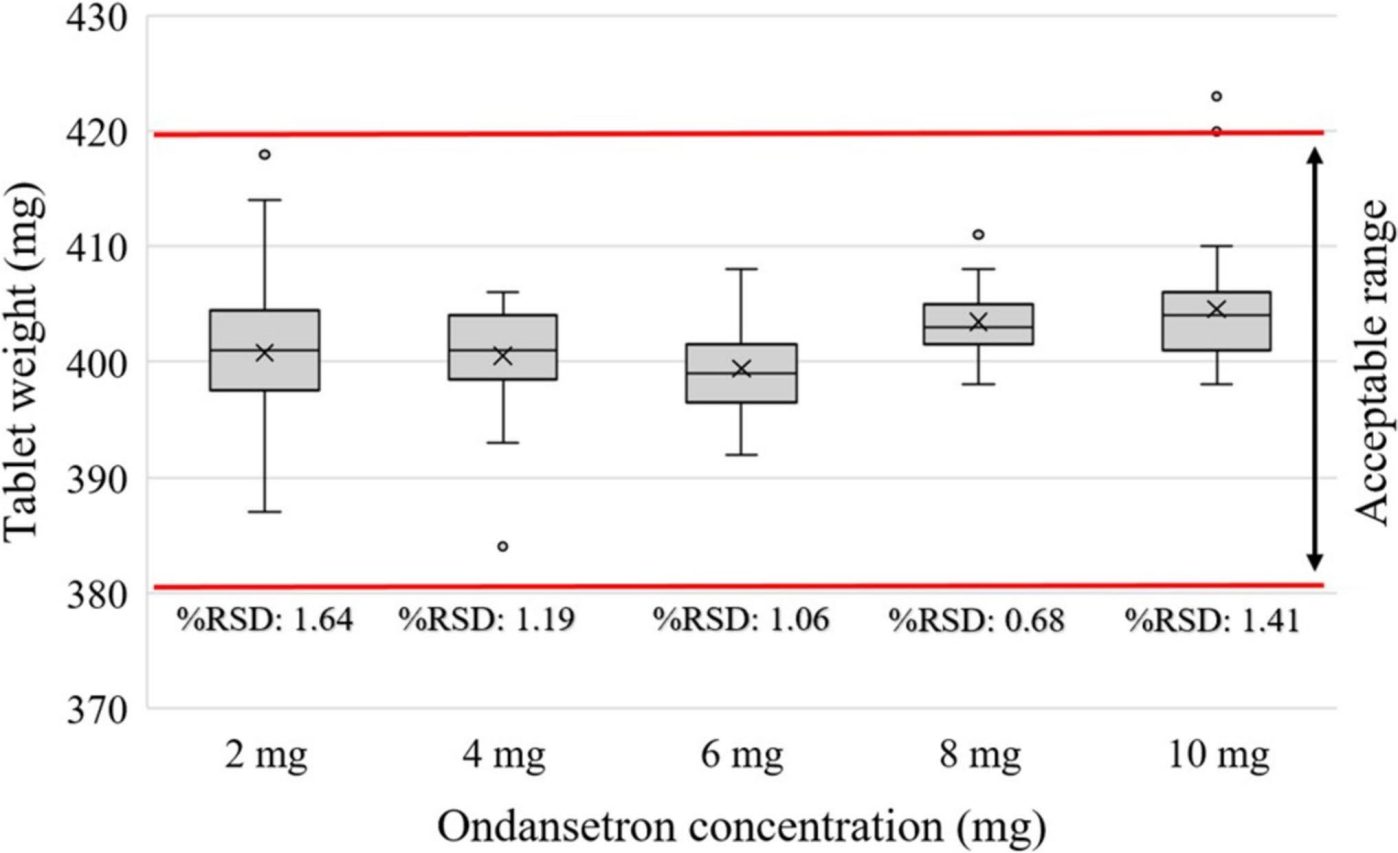
Mass uniformity results of different Ondansetron dosages with 400 mg tablet size

The mass uniformity results for different Ondansetron dosages with 400 mg tablet size showed that the average printing accuracy for 2, 4, 6, 8 and 10 mg Ondansetron were 100%, 100%, 100%, 100%, and 96% respectively. The box plot in Fig. 3 shows the distribution of tablet weights for different Ondansetron contents. Each box plot displays the median, interquartile range, and potential outliers. The %RSD values for each Ondansetron content are also provided in Fig. 3. The Tukey’s post hoc test revealed that there were no statistically significant differences in mass uniformity or printing accuracy among the 400 mg tablets containing 2, 4, 6, 8, and 10 mg of Ondansetron (P > 0.05 with the smallest p-value = 0.12 observed between 6 and 10 mg).

### Content Uniformity and Stability

The acceptance values for 200, 300, 400, and 500 mg tablets were determined to be 11, 8, 13, and 11 (Table III), respectively, while the limit according to the Ph.Eur. is AV < 15. Post hoc analysis using the Tukey’s test indicated a statistically significant difference in content uniformity between the 200 mg and 500 mg (P = 0.02) tablets, and 300 mg and 500 mg (P = 0.02) tablets containing 0.5% Ondansetron. In addition, the content uniformity of different Ondansetron content, i.e., 2, 4, 6, 8 and 10 conducted for 400 mg tablet size complied with the Ph.Eur. requirements (Table IV). The Tukey’s test showed significant differences in content uniformity between tablets containing 2 and 10 mg Ondansetron (P = 0.03), 4 and 10 mg Ondansetron (P = 0.01), and 6 mg and 10 mg Ondansetron (P = 0.01). In addition, Table V shows the stability of Ondansetron tablets up to 6 months storage in terms of pH, appearance and chemical stability of tablets at room temperature.

**Table III:** Content Uniformity Results for 0.5% Ondansetron Tablets with Different Tablet Sizes

Ondansetron content	Tablet Weight (mg)	Number of tablets	Minimum (%)	Maximum (%)	Mean (%)	%RSD	Acceptance value (AV)
0.5%	200	10	102	109	107	1.90	11
0.5%	300	10	101	111	107	2.80	13
0.5%	400	10	100	108	104	1.98	8
0.5%	500	10	95	108	103	3.62	11

**Table IV:** Content Uniformity Results for 2, 4, 6, 8, and 10 mg Ondansetron Tablets (400 mg)

Ondansetron dosage	Number of tablets	Minimum (%)	Maximum (%)	Mean (%)	%RSD	Acceptance value (AV)
2 mg	10	98	109	106	2.74	12
4 mg	10	100	109	107	2.24	11
6 mg	10	105	108	107	0.65	7
8 mg	10	103	107	106	1.23	7
10 mg	10	102	105	104	0.77	4

**Table V:** Stability Results for 0.5% Ondansetron Tablets

Test parameters (0.5% Ondansetron in CuraBlend® gel tablet base)	Zero-point	1 Month	3 Month	6 month
Appearance	off-white, soft, uniform color	off-white, soft, uniform color	off-white, soft, uniform color	off-white, soft, uniform color
Assay% (n = 6)	106.04%	105.95%	106.75%	105.79
pH (34- 44 °C)	4.87	4.88	4.90	4.90

### *In-vitro* Dissolution

**Fig. 4:**
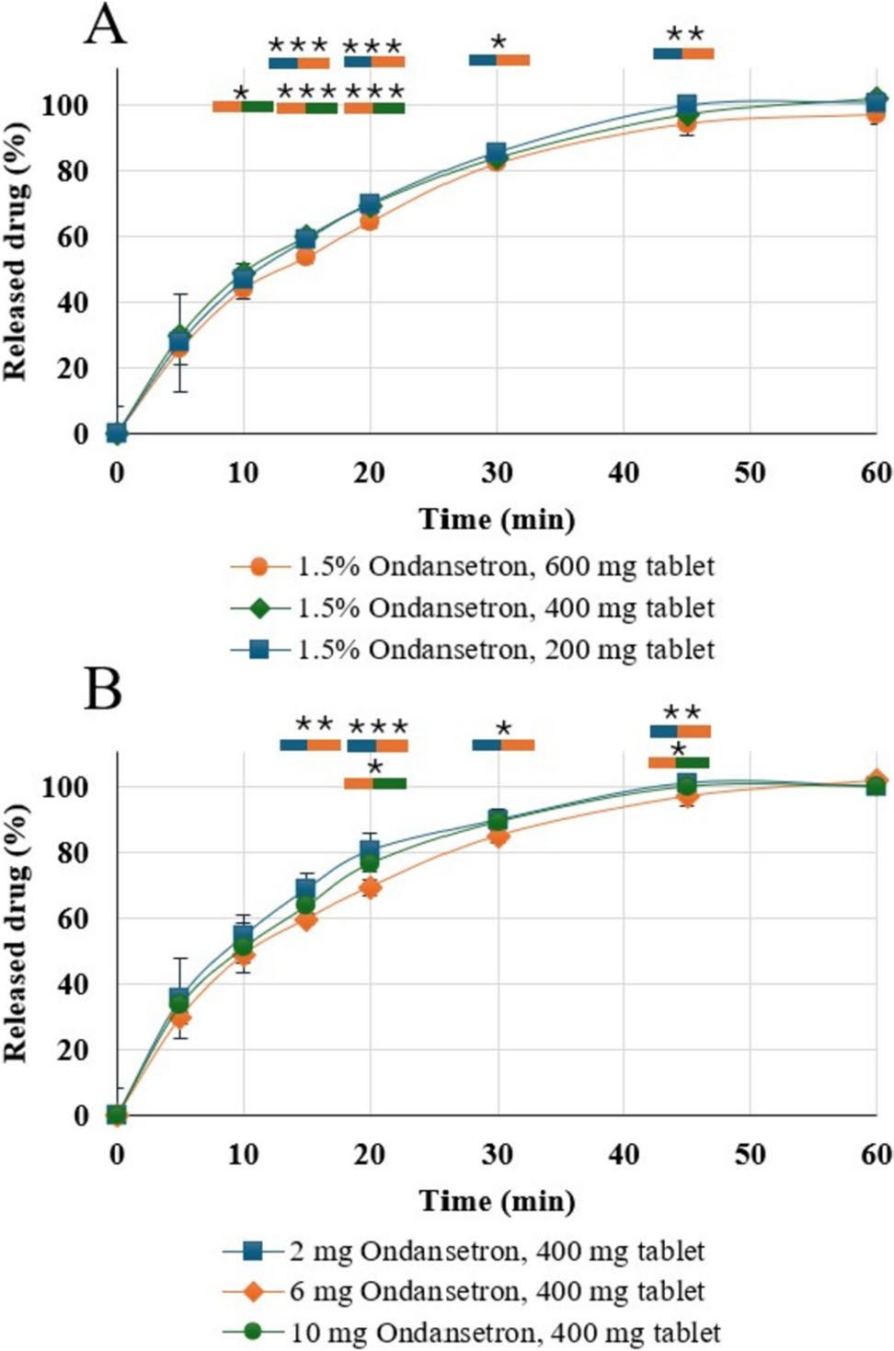
Dissolution profiles of 200 mg, 400 mg and 600 mg tablets containing 1.5% Ondansetron. B) Dissolution profiles of 400 mg tablets containing 2, 6 and 10 mg Ondansetron. Color bars indicate statistical significance differences between the groups (* p < 0.05, ** p < 0.01 and *** p < 0.001)

The effect of different tablet sizes (i.e., 200, 400 and 600 mg) containing 1.5% Ondansetron on drug release is shown in Fig. 4A. The dissolution profiles of 400 mg tablets containing 2 mg, 6 mg and 10 mg Ondansetron are shown in Fig. 4B. All Ondansetron dosages and tablet sizes released the API up to 85% within 30 min and about 100% within 45 min. Thus, all Ondansetron tablets complied with the requirements of the USP Pharmacopeial test 2, i.e., over 80% of the drug should be released within 30 min [30]. According to the results of the ANOVA test with Tukey's post hoc analysis, the 600 mg tablet demonstrated statistically significant differences in dissolution compared to the 400 mg tablet at 10, 15, and 20 min, and compared to the 200 mg tablet at 15, 20, 30, and 45 min. Similarly, among the 400 mg tablet formulations, those containing 2 mg and 6 mg of Ondansetron showed statistically significant differences in dissolution at 15, 20, 30, and 45 min. Additionally, a significant difference was observed between the 6 mg and 10 mg formulations at 20 and 45 min. Statistical significant differences are presented in Fig. 4.

### Quantitative Determination of Ondansetron Via RS

**Fig. 5:**
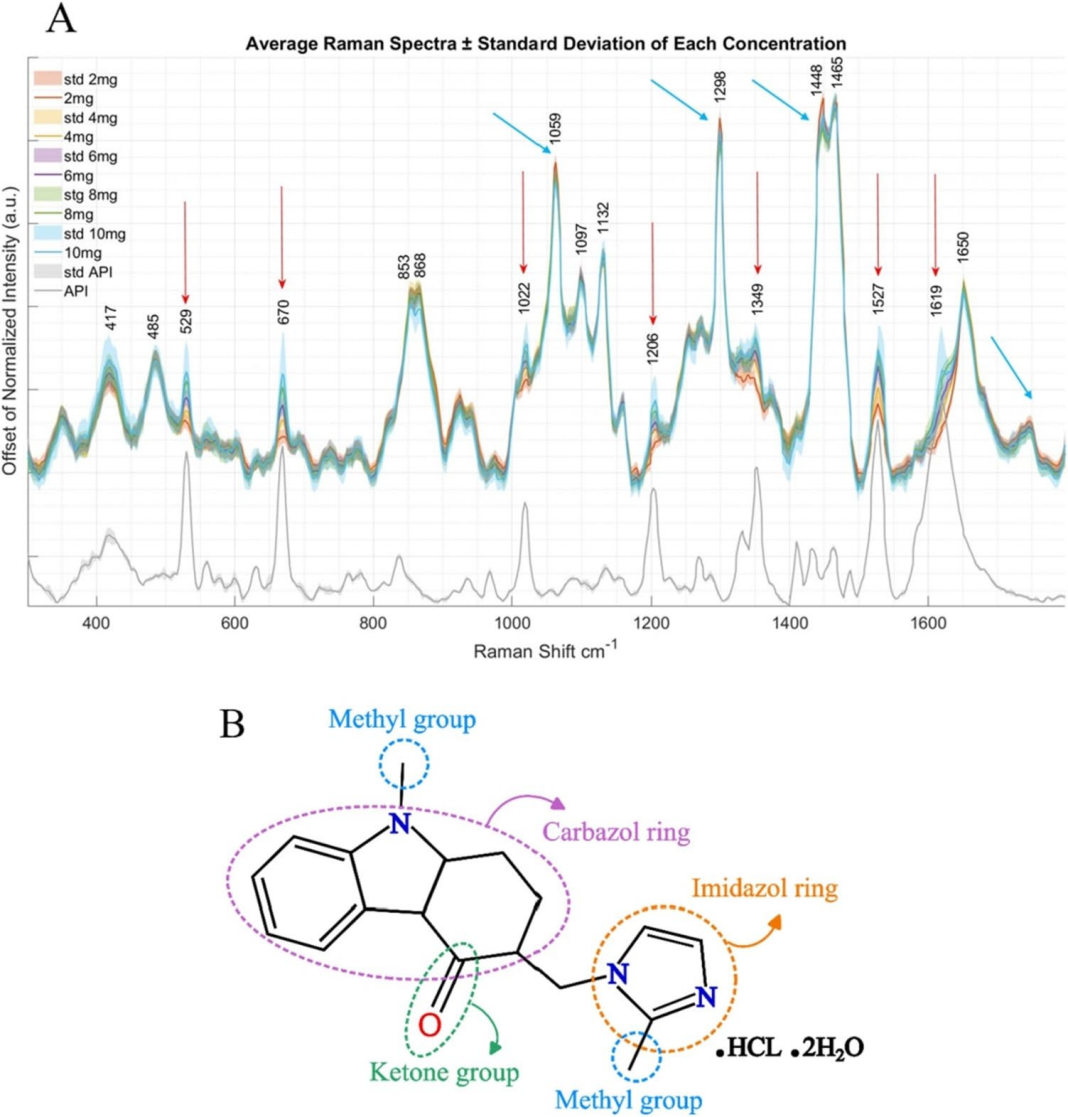
Averaged Raman spectra of 5 semi-solid extruded tablets containing 2, 4, 6, 8 or 10 mg Ondansetron, also including the spectrum of pure Ondansetron (API) in gray. Shaded areas indicate the standard deviation relative to 13 samples for each Ondansetron content. B) Chemical structure of Ondansetron highlighting each chemical ring or group that has a fingerprint in the Raman spectrum

Preprocessed Raman spectra of semi-solid extruded tablets containing different Ondansetron content and pure Ondansetron aqueous solution show clear differences which can be correlated to the API (Fig. 5A). The peaks correlating positively to Ondansetron are indicated by red arrows, while peaks correlating negatively with Ondansetron are indicated with cyan arrows. Visual analysis of the Raman spectrum of Ondansetron reveals prominent peaks at 529, 670, 1022, 1206, 1349, 1527, and 1619 cm^-1^ (Fig. 5A, red arrows). These peaks are also present in the semi-solid extruded Ondansetron tablets and show a positive correlation with the Ondansetron contents. Figure 5B shows the chemical structure of Ondansetron and highlights each chemical ring or chemical group that have a fingerprint in the Raman spectrum. Additionally, the Raman spectra of the semi-solid extruded Ondansetron tablets highlight features attributable to the excipients (Fig. 5A cyan arrows), such as the peak at 1059 cm^-1^ often linked to glycerol, the regions between 1230–1300 cm^-1^ (Amide III) and 1440–1470 cm^-1^ which are associated with lipid chains in cocoa butter, and the region between 1600–1700 cm^-1^ (Amide I) which is associated with proteins like gelatin. This underscores the viability of RS for monitoring the quality of semi-solid extruded tablets as it provides information on both the API and the excipients. The peak positions (cm^-1^) related to each chemical ring or chemical group in Ondansetron and chemical bonds in excipient compounds are shown in the Supplementary material Table S1.

**Fig. 6:**
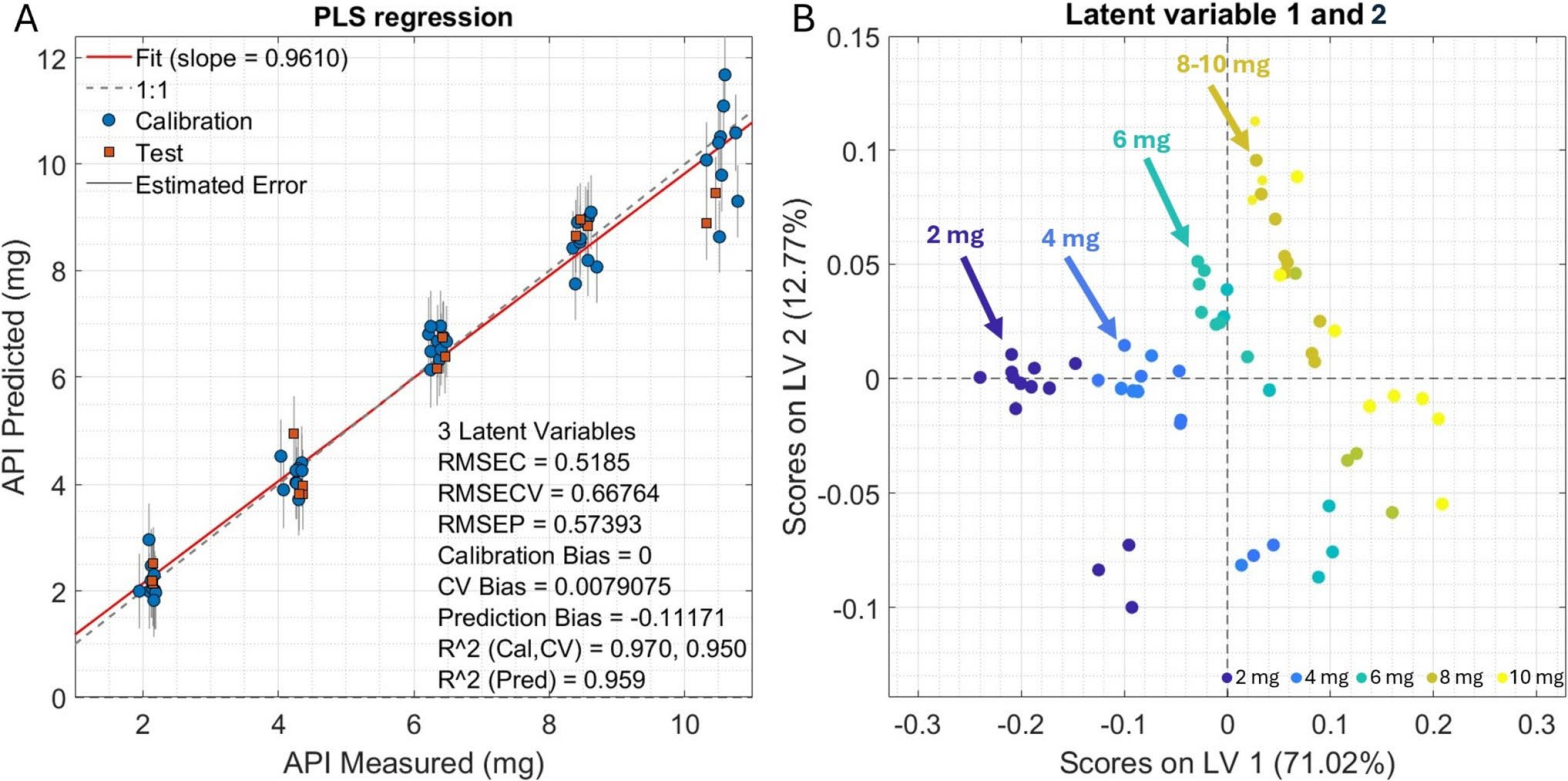
PLS regression and latent variable scores. (**A**) Blue dots indicate the training set, while the red squares indicate the sample set. Red line is the linear regression, while the black dashed line indicates the ideal line. (**B**) Scores on latent variable (LV) 1 and 2, color code blue to yellow) indicates the concentration from 2 to 10 mg

The performance of the PLS model is improved by removing variables with low Variable Importance in Projection (VIP), i.e., VIP < 0.8 and variables associated with high VIP score seems to be related to Raman peaks associated with Ondansetron (Supplementary material, Figure S2). The PLS regression results shown in Fig. 6A indicate a good correlation between the PLS prediction, and the Ondansetron content measured by HPLC both in terms of cross validation (R^2^CV, RMSECV) and prediction (R^2^ Pred, RMSEP). The R^2^ value for calibration, the CV and the prediction are close to 1 indicating that the predictor variables can explain ~ 87% of the variation in the response, while the deviation between the predicted Ondansetron quantity made by the model and the actual Ondansetron quantity is close to 0.6. The prediction bias, i.e., the difference between the estimated Ondansetron quantity and the reference Ondansetron quantity is higher than expected. This is particularly true for tablets with the highest Ondansetron content. Figure 6B shows the scores on LV1 and LV2. The clusters for 2, 4 and 6 mg are clear, while the clusters for 8 and 10 mg seems to overlap. The gray error bars in Fig. 6A for the 10 mg test samples appear larger, confirming higher uncertainty in prediction. This might be due to lack of reproducibility in the measurement method and the model could possibly be improved by including further measurements. Despite of this, the RS measurements and the PLS model shows high promise as a fast and non-invasive tool for monitoring the quality of semi-solid extruded tablets. For additional information see the supplementary materials.

## Discussion

The use of SSE-based CSS technology for manufacturing various tailor-made Ondansetron dosage forms demonstrates its capability for accurate and personalized drug production. This approach highlights the potential of SSE-based CSS in advancing individualized medicine. Moreover, quality control plays a crucial role in ensuring the safety, efficacy, and consistency of pharmaceutical products and dosage forms.

Personalized dosage forms of Ondansetron have recently been studied by Allahham and coworkers [12]. In their study, selective laser sintering (SLS) 3D printing (3DP) was used to fabricate orodispersible printlets (ODPs) to develop appropriate printed tablets containing Ondansetron. Ondansetron was mixed with cyclodextrin and various amounts of Mannitol. 3D printing of the developed formulation resulted in a fast-release product [12]. Mixing drugs with polysorbate can improve drug uniformity and aid in the disintegration of a solid dosage form [31, 32].

Polysorbate 80 was selected as the surfactant in the gelatin-based Ondansetron formulation because of its high solubilizing capacity and its ability to stabilize APIs and ingredients, thereby effectively preventing drug precipitation during the printing process. A concentration of 1% was chosen to ensure adequate solubilization while minimizing potential risks, particularly in neonates. Although there is no universal maximum limit exists for polysorbate 80 in pediatric formulations, its safe use is influenced by patient age, weight, and route of administration. Ondansetron contents ranging from 2–10 mg are commonly administered to pediatric patients with ages between 6 months to 11 years. According to [33], the Progressive Pediatric Safety Factor (PPSF) model provides a conservative, age- and weight-based framework for estimating safe excipient levels. Based on this model, the maximum allowable amount of polysorbate 80 for a 6-month-old infant is approximately 6 mg per day, which aligns well with the amount present in the formulations in this study that contained 2 mg of Ondansetron.

Furthermore, in other studies, different semi-solid base formulations have been used for 3D printed dosage forms. For example, an agar-HPMC-based matrix has been used as a 3D printing material for drug compounding [34]. Moreover, in another 3D printing study, PVA (Polyvinyl alcohol) was utilized as a base formulation and mixed with Paracetamol [15]. In addition, the SSE technology has also been investigated to produce caffein tablets for neonates by using a hydrogel-based formulation containing HPMC and agar [34]. In a more recent study, lipid Gelucire 50/30 was used as the main excipient in the semi-solid base formulation for 3D manufacturing of hydrocortisone tablets [14]. However, these Hydrocortisone tablets did not comply with the US Pharmacopoeia acceptance criteria of an immediate release drug dosage form. SSE 3D printed chewable tablets using a gelatin-based matrix for pediatric patients has been investigated during the last decade. In one study, propranolol hydrochloride gummy tablets were fabricated using SSE 3D printing and it was revealed during the formulation optimization process that gelatin and carrageenan significantly influenced printability, mechanical properties, and disintegration behavior, while γ-aminobutyric acid effectively masked bitterness to enhance pediatric compliance [35]. In addition to demonstrating the feasibility of SSE 3D printing for accurately producing single-drug dosage forms, this technology also holds promise for fabricating polypill tablets containing two or more APIs to support personalized therapy. For example, a recent formulation combined Isoniazid and Pyridoxine in a gel matrix containing gelatin, carrageenan, and xylitol to create a pediatric-friendly polypill for tuberculosis treatment, thus highlighting the potential of SSE 3D printing to improve medication adherence in children through dose personalization [36].

In this study, Ondansetron and Polysorbate 80 were mixed with a gelatin-based formulation, i.e., CuraBlend® gel tablet base, at elevated temperatures to achieve a uniformly blended matrix. The GMP-manufactured CuraBlend® gel tablet base consists mainly of purified water, xylitol, gelatin, cocoa butter, and pH adjusting agents, preservative and flavoring agents. Upon cooling, the formulation solidifies and can be reactivated into a semi-solid gel with mild heating, thereby enabling its use in SSE 3D printing. This semi-solid matrix serves as a printable base that can be easily dispensed into blisters and exhibits immediate *in vitro* drug release behavior.

Mass variation results of the different SSE Ondansetron tablets (i.e., different tablet sizes and Ondansetron contents) highlight the consistency of the printing process used in this study. A balance integrated into the Pharma Printer was employed to record the mass variation during tablet manufacturing. A similar approach was reported in a recent study, where an integrated balance and software system within the 3D printer recorded the target printed tablet mass. The balance measured the weight of each oral tablet and the software recorded the data [14]. According to the European Pharmacopeia (Ph. Eur. 2.9.5. Uniformity of mass for single-dose preparation-test), a deviation limit of ± 5% and ± 7.5% is applied to tablets weighing over 250 mg and under 250 mg, respectively. To investigate the influence of tablet size and API content on mass and content uniformity, a series of tablets containing Ondansetron were formulated and tested. Tablets of 200 mg, 300 mg, 400 mg, and 500 mg, each incorporating 0.5% Ondansetron (equivalent to 1–2.5 mg per tablet), were evaluated alongside 400 mg tablets containing varying Ondansetron content ranging from 2 to 10 mg. This design enabled a comprehensive assessment across a total API content range of 1–10 mg, which aligns with the therapeutic requirements for pediatric populations. The study aimed to determine whether variations in tablet mass and API content significantly affect the uniformity of dosage units, which is a critical quality attribute for ensuring consistent therapeutic efficacy and safety in pediatric patients [37].

Printing accuracy of 200 mg, 300 mg, 400 mg, and 500 mg tablets were 100%, 100%, 96%, and 99%, respectively. In addition, the printing accuracy for 400 mg tablets containing 2, 4, 6, 8, and 10 mg Ondansetron were 100%, 100%, 100%, 100%, and 96%, respectively. Moreover, in terms of tablet diameter and thickness, the lower RSD values observed in the 500 mg tablets compared to the 200 mg tablets can be attributed to the higher volume of formulation used. In the case of 500 mg tablets, the semi-solid formulation fills the entire blister cavity more uniformly during the printing process, which promotes consistent tablet shape and dimensions. In contrast, the smaller volume in the 200 mg tablets may not spread as evenly, which leads to greater variability in diameter and thickness measurements. From a statistical standpoint, no significant differences were observed between tablet sizes containing 0.5% Ondansetron, nor among the 400 mg tablets formulated with varying Ondansetron concentrations (P > 0.05). Nevertheless, the obtained results demonstrated the ability of the Pharma Printer to control the printing accuracy for Ondansetron tablets in different dosages and tablet sizes with high standard.

All acceptance values (AV) for content uniformity of the Ondansetron tablets prepared in this study were below 15, indicating compliance with pharmacopeial standards. Acceptance values under 15, combined with low standard deviations, reflect good uniformity of drug distribution within the tablets [29]. Notably, tablets with higher Ondansetron content exhibited lower AVs and mass uniformity standard deviations, which suggests that increased API content contributes to improved content uniformity and printability [38, 39]. The highest AV was observed for the 2 mg Ondansetron formulation in 400 mg tablet size and within the 300 mg tablet containing 0.5% Ondansetron groups. Although this formulation met the acceptance criteria, it may benefit from further optimization of the manufacturing process and formulation parameters. Statistical analysis revealed a significant difference in content uniformity between the 200 mg and 500 mg (P = 0.02) tablets, and 300 mg and 500 mg (P = 0.02) tablets containing 0.5% Ondansetron. Additionally, a significant difference was observed in content uniformity between tablets containing 2 and 10 mg Ondansetron (P = 0.03), 4 and 10 mg Ondansetron (P = 0.01), and 6 mg and 10 mg Ondansetron (P = 0.01). These findings suggest that larger tablet sizes and higher drug content are associated with improved content uniformity in SSE-printed Ondansetron tablets. This observation aligns with previous studies. For example, a recent investigation in the rheological behavior and printability of SSE formulations demonstrated that formulation composition including drug and excipient concentrations significantly affects printability and dimensional accuracy [16]. Specifically, formulations with lower API content were more prone to exhibit inconsistent flow and deposition, thus leading to greater variability in mass and content uniformity.

Furthermore, a study using the same CuraBlend® gel tablet base used in this study found that tablets with higher concentrations of Clopidogrel, particularly in the 500 mg size, exhibited superior printing accuracy and lower AVs, indicating enhanced printability and content uniformity [38]. Additionally, the larger tablet size (500 mg) formulated with Prednisolone and Spironolactone in the CuraBlend® gel tablet base have also demonstrated enhanced printing accuracy and content uniformity [19]. This is consistent with our observation that the 2 mg Ondansetron formulation exhibited the highest %RSD in printing accuracy and content uniformity. At such low API content, minor inconsistencies in mixing, extrusion pressure, or rheological behavior can have a disproportionately large effect on the final dosage form.

The six-month stability study of 0.5% Ondansetron tablets revealed consistency in both visual and chemical properties. Throughout the study period, the tablets retained their off-white color, soft texture, and uniform appearance, indicating no observable changes in physical appearance visually. The assay results for Ondansetron in the tablets up to six months of storage fall within the acceptable limits based on ICH Q1A (R2) guidelines [40] This shows that the active ingredient remains stable and potent over the six-month storage period. Additionally, pH values showed minimal variation, ranging from 4.87 to 4.90, which supports the reproducibility, effectiveness, and safety of the formulation upon administration [41, 42].

The dissolution profile of the semi-solid tablets was evaluated over 60 min. This study investigated two variables: (1) the effect of tablet size (200, 400, and 600 mg) with a constant drug content of 1.5% Ondansetron, and (2) the effect of varying Ondansetron content (2, 6, and 10 mg) within a fixed 400 mg tablet size. The Tukey’s post hoc analysis showed that the 600 mg tablet had significantly different dissolution drug release (slower) compared to 400 mg and 200 mg tablets. Among the 400 mg tablets, differences were also found between formulations with 2, 6, and 10 mg of Ondansetron in 15, 20, 30, and 45 min time points (Fig. 4). These findings are consistent with recent studies suggesting that drug release is influenced by the surface area-to-volume ratio of the dosage form. Tablets with a higher relative surface area (i.e., smaller volume) tend to release the drug more rapidly than larger tablets with a lower surface area-to-volume ratio [14, 15, 35]. Overall, the dissolution profile demonstrates that the Ondansetron tablets meet the criteria for an immediate-release solid oral dosage form, with not less than 80% of the drug released within 30 min for all six tablets tested. Therefore, the dissolution results comply with the requirements outlined in Ph. Eur. 2.9.3 and Ph. Eur. 5.17.1. Furthermore, the results also satisfy the specifications for Ondansetron oral tablets according to the USP Pharmacopoeia Test 2, which similarly requires not less than 80% drug release within 30 min [30].

RS has already been employed as a non-destructive quality control tool for 3D printed tablets [25, 26, 43]. This technology, for the case presented in this study, shows practical advantages such as its insensitivity to water (CuraBlend® gel tablet base formulation has significant amount of water) which usually gives a strong IR signal and can in some cases overwhelm the analyte signals. [44]. In addition, RS has recently been used for detecting paracetamol distribution in a base formulation [45], identify raw materials, food manufacturing [46], drug impurity monitoring over stability time-points [43, 47], API quantifications [24, 48], and drug expiry monitoring [27].

The integration of RS into pharmaceutical analysis represents a powerful tool for non-destructive, rapid, and precise quantification of APIs. In this study, Partial Least Squares (PLS) regression was used to model the relationship between spectral data (RS spectrum) and API concentration (HPLC data). For model development and validation, a total of 65 averaged Raman spectra derived from five replicate measurements per tablet were used. The dataset of 65 samples was divided into two sets: a training set, with 50 samples and a test set with 15 samples. Thus, ~ 75% of the samples were used for training and ~ 25% for testing to balance the need for a sufficiently large training set to build a robust and generalizable model, while retaining enough independent samples to reliably assess predictive performance and avoid overfitting. 2–4 random samples for each concentration were chosen for the test set. In doing so, there were 50 samples for the training set which was enough to use a continuous block with block size of 10 measurements as cross-validation.

The coefficient of determination (R^2^), which reflects how well the model explains the variability in the data, showed high values: 0.970 for calibration, 0.950 for cross-validation, and 0.959 for prediction. This usually means that the PLS model fits the training data better than it generalizes to new, unseen data, which is normal and expected. The Root Mean Square Error (RMSE), a measure of the average prediction error, was low across calibration (0.5185), cross-validation (0.6676), and prediction (0.5739), suggesting high predictive accuracy. This is consistent also in the case of the RMSE, where RMSE calibration < RMSE validation, which means that the model performs better on training data than on new data, which is expected. Bias values were near zero, suggesting that there is no systematic over- or under-prediction, which is good.

Additionally, Variable Importance in Projection (VIP) scores used to identify which spectral variables influenced the model most are essential for understanding which molecular features (vibrational modes of chemical bonds in the drug structure) drive the predictions. Notably, in case of Ondansetron, prominent peaks at 529, 670, 1022, 1206, 1349, 1527, and 1619 cm^-1^ were identified as key contributors, indicating that these wavenumbers represent structurally or functionally significant regions of the molecule that the model relies on for accurate quantification. For pharmaceutical scientists, these metrics provide a practical framework for evaluating the robustness and reliability of Raman-based models in quality control and formulation development.

Latent variables (LV) are some variables presumed to exist but not directly observed. LV1, which explains 71% of the variance, seems the one directly associated to the concentration of the API. LV1 (figure S5) have 6 main positive peaks (530, 670, 1206, 1340, 1530 and 1620), some of which are related to the API. Furthermore, LV2 increases as a function of Ondansetron concentration. On the other hand, LV2 is also affected by signals in the 1440 region (figure S5), which is the C-H area reflecting the total organic carbon content. Thus, the variability in this area reflects the overall intensity of the signal. Consequently, LV1 seems more capable to discriminate samples at low concentration, while LV2 discriminate samples with higher concentrations.

The plot of LV1 and LV2 scores (Fig. 6B) shows that the clusters for 2, 4 and 6 mg are very distinct, while the clusters for 8 and 10 mg seems to overlap. However, one of the reasons for the large error in the PLS model is due to the high variability of the 10 mg Ondansetron samples and could possibly be reduced by increasing the number of samples or alternatively use a more stringent outlier selection criterion. Furthermore, other reasons that the prediction relative to the 10 mg tablets shows a higher variability compared to the other samples might originate from the lack of reproducibility of the measurement method, i.e., changes in distance of measurement between the sample and the RS probe or different rotational speed of the sample holder. In addition, there is scientific support for the idea that non-linear relationships between spectral intensity and analyte concentration can occur at higher API loads, particularly in semi-solid matrices [49]. At higher drug concentrations, the Raman signal response may become non-linear due to saturation effects, increased scattering, and matrix-induced variability, particularly in semi-solid formulations. These phenomena can compromise the accuracy of chemometric models like PLS, as is also noted in the recent RS literature [50]. Assessing and developing these issues would most probably improve the performance of the PLS model, and consequently improve its prediction accuracy. Nevertheless, although the RS measurements and the PLS model developed in this study for Ondansetron quantification would still require some optimization, PLS models based on RS measurements shows high promise as fast and non-invasive tools for monitoring the quality of semi-solid extruded tablets, even for tablets with low amounts of API.

## Conclusions

The results of this study demonstrate that the SSE-based CSS approach holds significant promise for simplifying and automating the personalized manufacturing of pediatric pharmaceuticals. The successful preparation of tablets with varying Ondansetron contents and tablet weights highlights the versatility and precision of this method. The high dosing accuracy of the Pharma Printer, excellent API uniformity, good storage stability in the gelatin-based matrix, and immediate-release dissolution behavior collectively underscore the potential of SSE technology in developing stable, effective, and personalized dosage forms. RS complements the SSE-based CSS approach by providing a non-destructive and accurate quality control method that enhances dosing precision and ensures the safety and efficacy of personalized medicines. This study demonstrates the feasibility of integrating SSE technology with RS as a robust platform for producing tailored pharmaceutical solutions for pediatric patients. However, further development is needed to fully integrate SSE-based compounding systems with next-generation quality control technologies such as RS. Continued efforts should focus on improving the accuracy, robustness, and repeatability of these systems to meet the stringent requirements of routine use in pharmaceutical manufacturing environments.

## Supplementary Information

Below is the link to the electronic supplementary material.

Supplementary file1 (DOCX 6787 KB)

## Data Availability

This is not applicable since the data is a property of the CurifyLabs company.

## Abbreviations

HPLC: High-Performance Liquid Chromatography
QCA: Quality Control Analysis
SSET: Semi-Solid Extruded Tablets
API: Active Pharmaceutical Ingredient
LOD: Limit of Detection
LOQ: Limit of Quantitation
LV: Latent variable
RSD: Relative Standard Deviation
CV: Cross validation
AV: Acceptance Value
HPMC: Hydroxypropyl Methylcellulose
PLS: Partial Least square
RMSE: Root Mean Square Error
RMSEP: Root Mean Square Error Prediction
RMSECV: Root Mean Square Error Cross validation
RS: Raman Spectroscopy
SD: Standard deviation
SSE: Semi-Solid extrusion
PS80: Polysorbate 80
3DP: Three-dimensional printing
CAD: Computer-aided design
CSS: Compounding System Solution
RT: Retention time
