# Supplementary material for: Assessment of a Semi-solid Extrusion Based Compounding System Solution for Personalized Ondansetron Dosage Forms Combined with Raman Spectroscopy Analysis
Source: Pharm Res. 2025 Aug 19;42(9):1631–45. doi: 10.1007/s11095-025-03911-6 (PMC12507975; doi:10.1007/s11095-025-03911-6)
Supplement: Supplementary file 1 — Supplementary file1 (DOCX 6787 KB) [file 11095_2025_3911_MOESM1_ESM.docx]

Supplementary materials

Assessment of a Semi-Solid Extrusion Based Compounding System Solution for Personalized Ondansetron Formulation Combined with Raman Spectroscopy Analysis

Mahsa Bahman^1, 2^, Jacopo Zini^3^, Julius Lahtinen^2,4^, Niko Hassinen^2^, Soumya Verma^2^, Timo Laaksonen^3^, Sari Airaksinen^2^, Niklas Sandler Topelius^1,2^, Tapani Viitala^1,4^

^1^Pharmaceutical Sciences Laboratory, Åbo Akademi University, Artillerigatan 6A, 02520 Turku, Finland

^2^CurifyLabs Oy, Salmisaarenaukio 1, 00180 Helsinki, Finland

^3^ Drug Research Program, Division of Pharmaceutical Biosciences, Faculty of Pharmacy, University of Helsinki, Viikinkaari 5E 00790 Helsinki, Finland

^4^ Drug Research Program, Division of Pharmaceutical Chemistry and Technology, Faculty of Pharmacy, University of Helsinki, Viikinkaari 5E 00790 Helsinki, Finland

**HPLC method development and validation**

**Analytical method development**

The HPLC method was developed to conduct long-term stability test, content uniformity test, and in-vitro dissolution test to ensure about the suitability of printed Ondansetron tablets manufactured by the Pharma Printer. HPLC method development was performed based on physical and chemical properties of Ondansetron. For this purpose, several parameters such as the mobile phase, pH, flow rate, and column temperature were tested to achieve the best separation and peak resolution. For HPLC analysis of Ondansetron, potassium phosphate monobasic (Sigma Aldrich Steinheim, Germany) and acetonitrile (Fisher Scientific, Loughborough, UK) (70:30), in isocratic flow, and pH adjusted by phosphoric acid (ACS reagent, Fisher Scientific, Waltham, Massachusetts, USA), were used as mobile phases. An HPLC (Thermo Scientific™️ Vanquish system, Germering, Germany) with the Chromeleon™️ Chromatography Data System software (Dionex Softron GmbH, Germering, Germany) was utilized for sample analysis. The HPLC system was equipped with a C18 column (4.6 × 100 mm i.d., 2.5 μm particle size, VanGuard FIT, Wilmslow, UK) and Diode Array Detectors (Thermo Scientific Vanquish detector, Dionex Softron GmbH, Germering, Germany).

**Analytical method validation**

The selected HPLC analytical method was validated according to ICH Q2 (R1) guidelines (“Validation of analytical procedures Q2 (R2),” 2023). The method validation parameters such as, linearity, accuracy, precisions, robustness, LOD, and LOQ were determined to assess whether the developed analytical method is reliable, accurate, and suitable for its intended purpose.

**Discussion on HPLC method development and validation**

A satisfactory HPLC analysis method and a good peak symmetry was achieved by using a C18 column as a stationary phase with a mobile phase containing 20 mM phosphate buffer and Acetonitrile in a ratio of 70:30 v/v at a flow rate of 0.7 ml/min. The detection wavelength used was 216 nm. The retention time was at 2.8 and the asymmetry factor was 1.5 when sample volume injection was 10 µL.

The R^2^ for the linear fit of the Ondansetron HPLC method was R^2^ > 0.99 confirming a good linearity of the method. Percentage of recovery or accuracy test for 70% (35 µg/ml), 100% (50 µg/ml), and 130% (65 µg/ml) Ondansetron was 99.5%, 99.3%, and 100.7%, respectively. Thus, demonstrating a good accuracy for the method. The difference in average results of intermediate precision test of 70%, 100%, and 130% spiked samples, i.e., API + placebo, between two analysis days was observed to be less than the required 2% for a good, validated method, i.e., 0.39%, 0.17%, and 0.01%, respectively. Moreover, the %RSD values between six aliquots of the target standard of 50 ppm (100%) as an inter-day precision was less than the required 2%. The robustness data showed that the HPLC method is sensitive in respect to flow rate changes (±10% ml/min) since the analyte spends more time in the detector when using a lower flow rate, which leads to a larger peak area compared to a higher flow rate. Thus, the flow rate must be kept constant. However, the HPLC method showed to be robust with regards to column oven temperature changes (±5 °C) and different column lot numbers. According to the achieved validation results, the developed HPLC method for Ondansetron in this work can be applied for routine quality control analysis. The sensitivity of the method was tested by measuring the LOD and LOQ, and these were found to be 0.0625 µg/ml and 0.125 µg/ml, respectively.

In summary, an HPLC method for Ondansetron detection using a combination of phosphate buffer and acetonitrile (70:30 v/v) was successfully developed and validated. The validation of this HPLC method was conducted according to ICH (Q2) guidelines [34]. Based on ICH guidelines, the developed HPLC method was validated for accuracy, linearity, inter and intra-day precisions, robustness, LOD and LOQ. All other parameters for the HPLC method complied with the ICH guidelines except the robustness.

**Raman Spectroscopy figures and data**

**Process for PLS regression**

The collected spectra were smoothed and mean centred before PLS regression (Figure S1). Variables where selected based on their VIP value and the variables selected are highlighted in Figure S*2*. Two samples were considered as outliers and removed (Figure S3) due to the high residuals and Hotelling score. Three LV:s were selected for the regression. This choice is based on the RMSE and variance captured shown in Figure S4A and S4B. Three LV:s have lower RMSEP compared to four LV:s and the RMSECV levels out at three LV:s. Additionally, the three LV:s explain 86.4% of the cumulative variance, while the following LV would only add little expanded variance (≤ 2%) (Figure S5).


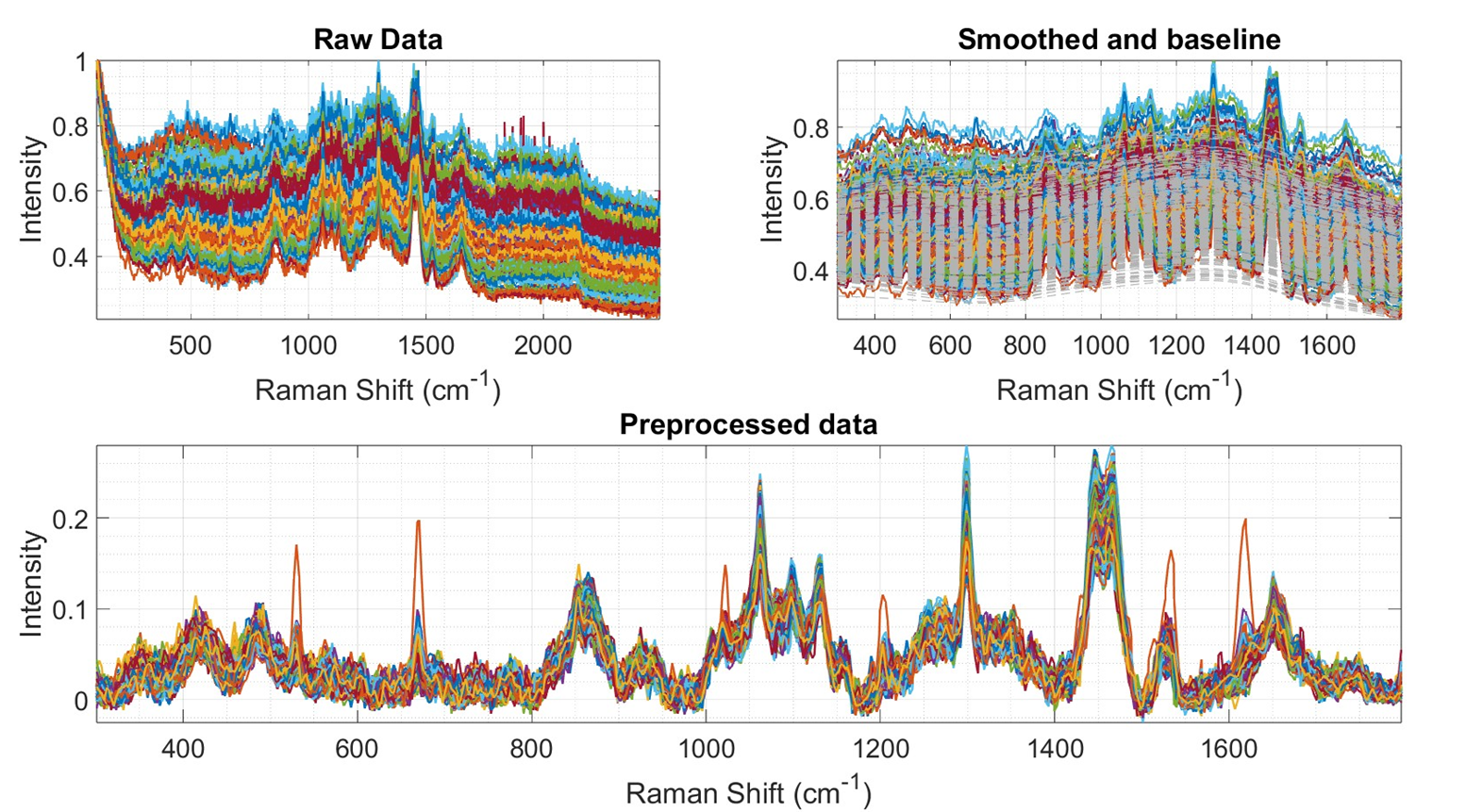


A

B

C

Figure S1. Preprocessing of Raman spectra. A) Raw data, B) Smoothed spectra and baseline (in grey), C) Baseline corrected spectra. These spectra were mean centered before PLS regression.


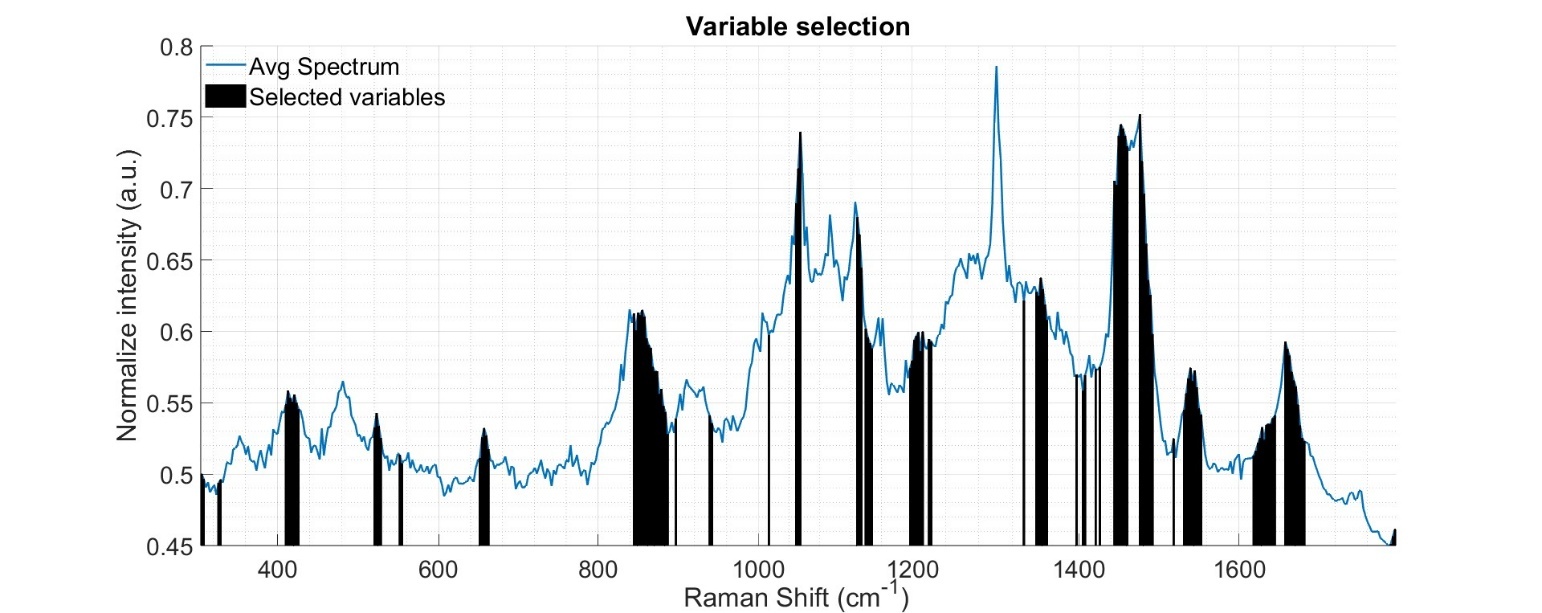


Figure S2. Variable selection: variables were selected based on their Importance in Projection (VIP) scores which estimate the importance of each variable in the projection used in a PLS model. The blue line indicates the average of the spectra, while the black areas indicate the selected variable used for the PLS model.


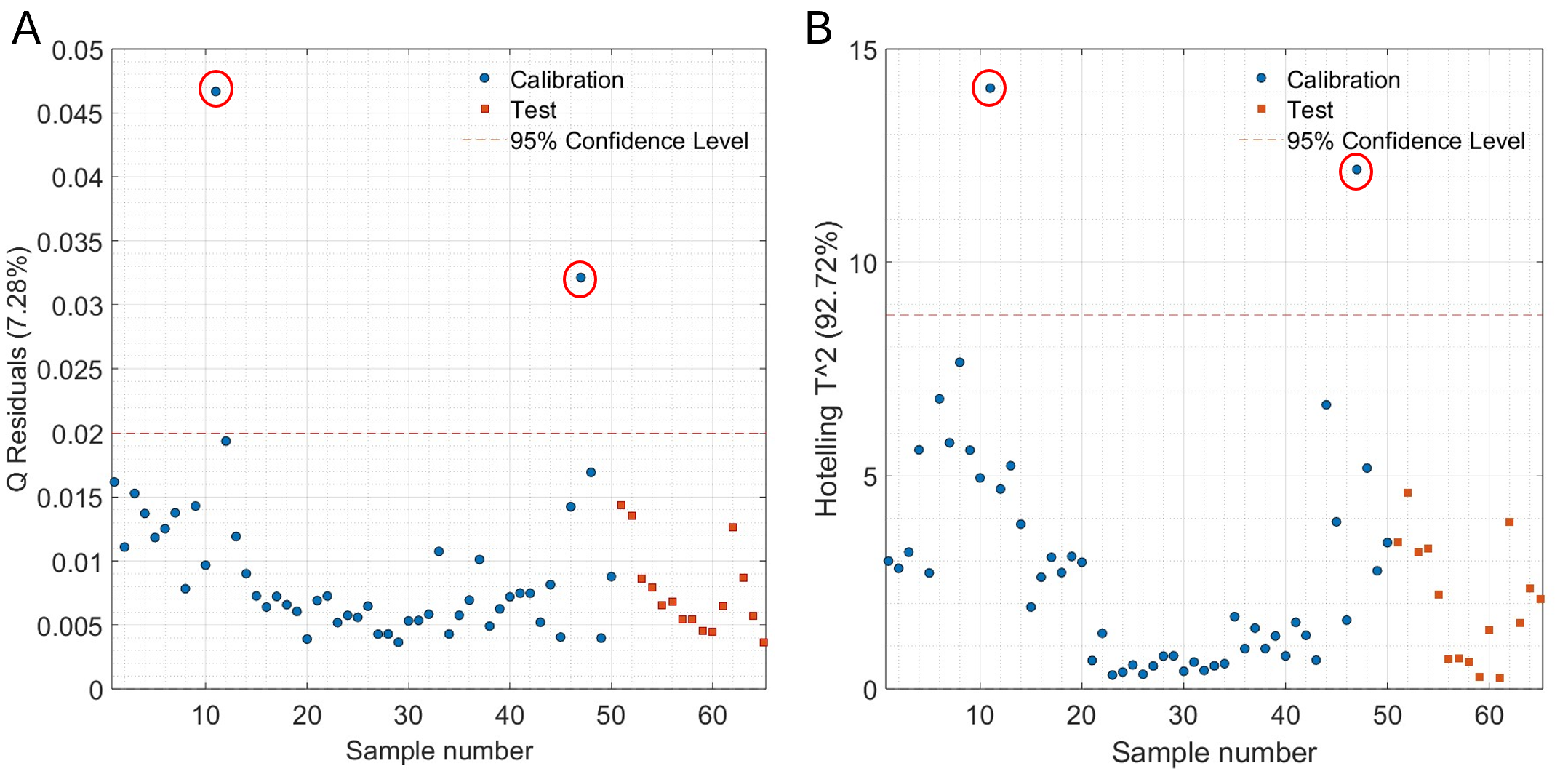


Figure S3. Outlier exclusion. PLS regression on the 65 samples indicate two outliers, i.e., samples 11 and 47, which both display A) a high Q residual and B) a high Hotelling T^2.


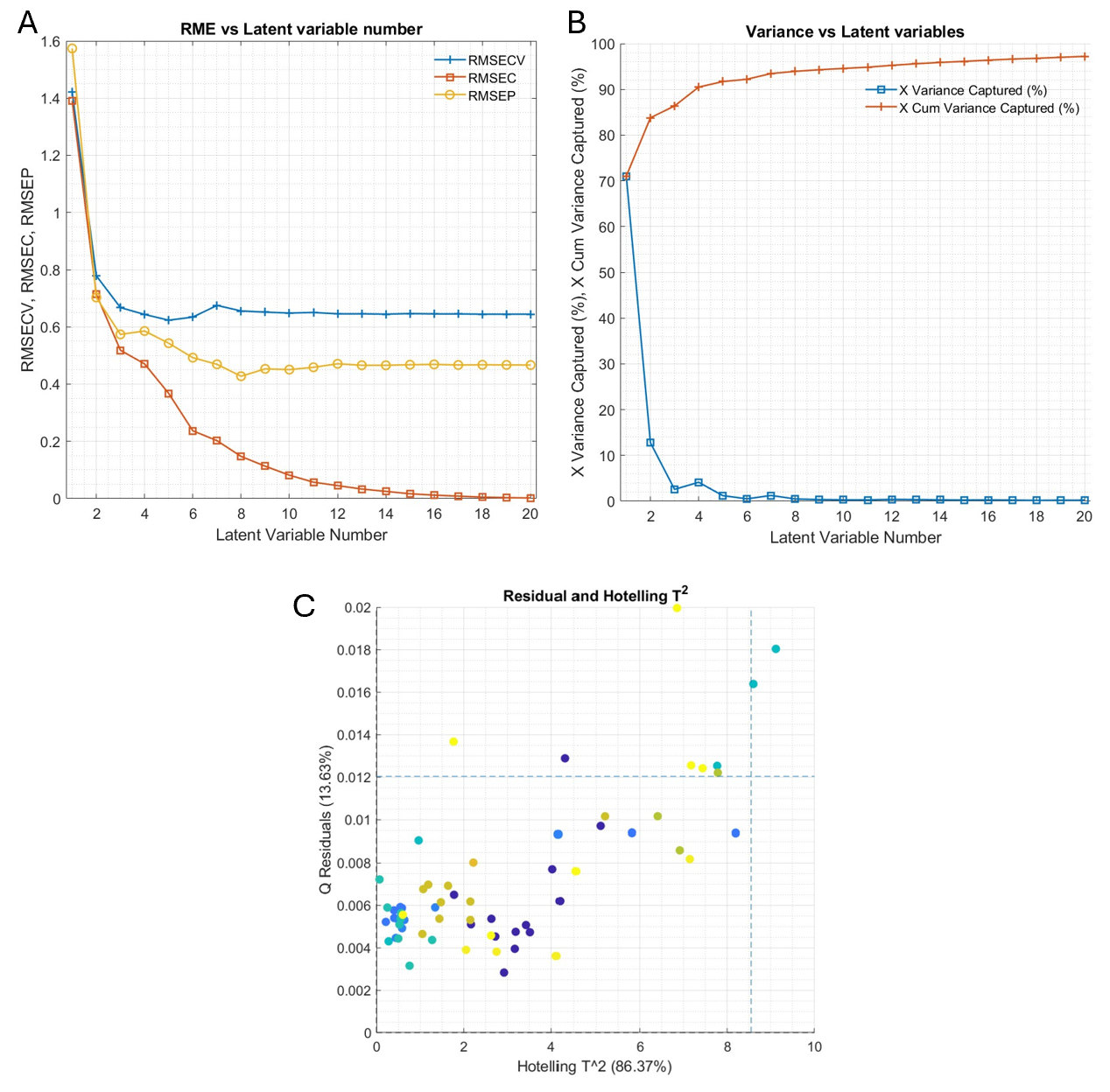


Figure S4. PLS regression statistics. A) RMSE for calibration, cross-validation and prediction based on number of variables. B) Variance captured, C) Hotelling T^2 and residual for each sample.

.


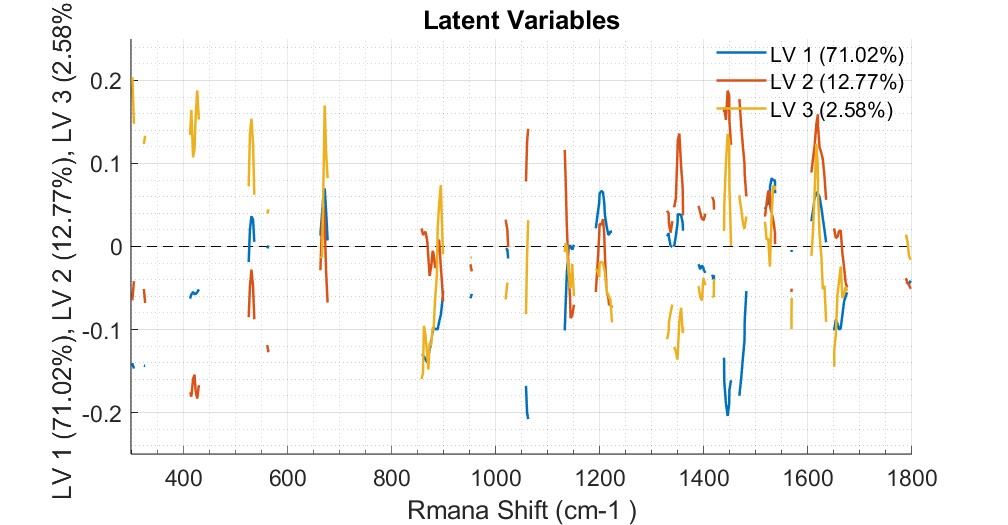


Figure S5. Latent variables. Plot of the LV 1 (blue), LV 2 (red) and LV 3 (yellow) used for the PLS regression.

Table S1. tentative Raman peak assignments and their relations with the semi-solid extruded tablet components. Abbreviations: deformation, def.; stretching, str.; rocking, rock.; bending, bend.; scissoring, sciss.

| Peak position (cm^-1^) | Chemical bond | Compound |
| --- | --- | --- |
| 485 | Si-O-Si | Silica, excipient |
| 529 | C-N-C | Ondansetron, Imidazole ring, tertiary amine nitrogen |
| 670 | (C=O)-C def. | Ondansetron |
| 853 | various vibration C-C | Aromatic amino acids, gelatin, excipient |
| 868 | various vibration C-C,  C-C str, CH_2_ tw, ring def, | Aromatic amino acids, gelatin, excipient  Indole (pyrrole) and Imidazole ring, Ondansetron |
| 1022 |  |  |
| 1059 | C-O str. | Glycerol and other sugars, excipient |
| 1097 | C-O-C def. | Glycerol and other sugars, excipient  Amino acids, gelatin, sugars excipient |
| 1132 | NH (term) rock., H-C-C bend. |  |
| 1206 | C-C-H ring, C-C, def | Indole, Ondansetron |
| 1298 | C-H def, O–H band. | Sugars, excipients |
| 1349 | N-CH, def. | Aromatic rings, Ondansetron |
| 1448 | CH_2_/CH_3_ bend. | Aliphatic chains of lipids, excipients |
| 1465 | CH_2_/CH_3_ sciss. | Aliphatic chains of lipids, excipients  Ondansetron |
| 1527 | Aliphatic azo |  |
| 1619 | C=C str, C=O def | Ondansetron |
| 1650 | Amide I | Gelatin, excipient |

**References:**

Validation of analytical procedures Q2 (R2) [WWW Document], 2023. URL https://www.ema.europa.eu/en/ich-q2r2-validation-analytical-procedures-scientific-guideline (accessed 12.18.24).
